# Comparison of LED therapy and low laser therapy in post-periodontal healing

**DOI:** 10.6026/973206300221220

**Published:** 2026-02-28

**Authors:** Pooja Mishra, Chavi Bhati, Akshay Ashok Katara, Sumeet Kaur, Rajeev Pathak, Deepak Thomas, Mehul A Shah

**Affiliations:** 1 Department of Periodontology and Oral Implantology, Kalka Dental College, Meerut, Uttar Pradesh, India; 2 Department of Dentistry, Omni Family Health, Bakersfield, California, United States; 3 Department of Dental Surgery, Hindu Rao Hospital, Malkaganj, Delhi, India; 4 Department of Periodontology and Oral Implantology, Teerthankar Mahaveer Dental College and Research Centre, Moradabad, Uttar Pradesh, India; 5 Department of Periodontics, Educare Institute of Dental Sciences, Malappuram, Kerala, India; 6 Department of Public Health Dentistry, Government Dental College and Hospital, Chhatrapati Sambhajinagar (Aurangabad), Maharashtra, India

**Keywords:** Light emitting diode (LED) therapy, low-level laser therapy (LLLT), periodontal surgery, photobiomodulation (PBM), wound healing

## Abstract

Postoperative pain, inflammation and delayed healing continue to be significant concerns following periodontal surgical procedures. Therefore, it is of interest to compare the effects of light emitting diode therapy and low-level laser therapy on post periodontal surgical healing. Hence, a total of 100 patients undergoing periodontal flap surgery were randomly allocated to receive either LED therapy or low-level laser therapy and healing outcomes were clinically evaluated. Both therapies significantly enhanced postoperative healing, with low-level laser therapy demonstrating superior early pain control and faster inflammatory resolution. This study advances existing knowledge by offering direct comparative evidence on photobiomodulation modalities for optimizing periodontal surgical healing.

## Background:

Periodontal surgical procedures, including flap surgeries, regenerative interventions and mucogingival surgeries, are routinely performed to arrest disease progression and restore periodontal health [1]. Despite advances in surgical techniques and biomaterials, postoperative inflammation, pain, delayed wound healing and patient discomfort remain significant clinical challenges. Optimal healing of periodontal tissues is critical for achieving long-term treatment success, as inadequate or delayed healing can compromise clinical outcomes, patient satisfaction and overall periodontal stability [2]. Consequently, adjunctive therapeutic modalities that can enhance wound healing and reduce postoperative morbidity have gained increasing attention in contemporary periodontal practice [3]. Photobiomodulation therapy (PBMT) has emerged as a non-invasive, chairside adjunct capable of modulating cellular and molecular processes involved in tissue repair. Among the various PBMT modalities, low-level laser therapy (LLLT) has been extensively studied for its biostimulatory effects on soft and hard tissues. LLLT is believed to enhance mitochondrial activity, increase adenosine triphosphate (ATP) production, promote fibroblast proliferation, stimulate angiogenesis and modulate inflammatory mediators, thereby accelerating wound healing and reducing pain [4]. Several studies have reported favorable outcomes of LLLT in periodontal surgery, including improved epithelialization, reduced edema and enhanced collagen synthesis. However, the clinical application of LLLT is limited by factors such as high equipment cost, the need for strict safety protocols and operator-dependent variables related to wavelength, energy density and exposure time [5]. Light emitting diode (LED) therapy has recently been proposed as an alternative photobiomodulation modality with comparable biological effects. LED devices emit non-coherent, monochromatic or quasi-monochromatic light within a specific wavelength range and offer advantages such as lower cost, ease of use, broader treatment coverage and minimal safety concerns [6]. Experimental and clinical evidence suggests that LED therapy can stimulate cellular metabolism, enhance microcirculation and regulate inflammatory responses in a manner similar to laser therapy [7]. In periodontal and oral surgical contexts, LED therapy has shown potential in reducing postoperative pain, accelerating soft tissue healing and improving patient comfort. Nevertheless, the non-coherent nature of LED light raises questions regarding its depth of penetration and overall efficacy when compared with coherent laser sources [8]. Although both LED therapy and LLLT are grounded in the principles of photobiomodulation, direct comparative evidence regarding their effectiveness in post periodontal surgical healing remains limited and inconclusive. Existing studies often vary in methodology, treatment parameters, surgical procedures and outcome measures, making it difficult to draw definitive clinical recommendations [9, 10]. Therefore, it is of interest to describe the comparative effectiveness of light emitting diode therapy and low-level laser therapy in promoting optimal post-periodontal surgical healing.

## Methodology:

The present study was designed as a prospective, randomized, controlled clinical trial to comparatively evaluate the effects of light emitting diode (LED) therapy and low-level laser therapy (LLLT) on post periodontal surgical healing. The study was conducted in the Department of Periodontology after obtaining approval from the Institutional Ethics Committee and all procedures adhered to the principles outlined in the Declaration of Helsinki. Written informed consent was obtained from all participants prior to their inclusion in the study. A total of 100 systemically healthy participants diagnosed with chronic periodontitis and requiring periodontal flap surgery were enrolled. The sample size was determined to provide adequate statistical power to detect clinically significant differences in postoperative healing outcomes between the two groups. Patients aged between 25 and 55 years, with comparable periodontal defects and satisfactory oral hygiene following initial periodontal therapy, were included. Exclusion criteria comprised the presence of systemic diseases affecting wound healing, such as diabetes mellitus or immunocompromised conditions, pregnancy or lactation, smoking habits, history of periodontal surgery within the previous six months and the use of medications known to influence inflammation or tissue repair. All participants underwent Phase I periodontal therapy, including scaling and root planing, along with standardized oral hygiene instructions. After a four-week revaluation period, eligible patients were randomly allocated into two equal groups of 50 participants each using a computer-generated randomization method. Group I received adjunctive LED therapy, while Group II received adjunctive low- level laser therapy following periodontal surgery. Periodontal flap surgery was performed under local anesthesia by a single experienced periodontist to minimize operator-related variability. The surgical protocol was standardized for all patients and included full-thickness mucoperiosteal flap elevation, meticulous debridement of the surgical site, thorough root surface instrumentation and flap repositioning with interrupted sutures. In Group I, LED therapy was administered using a commercially available LED device emitting light within the red wavelength spectrum. The light source was positioned at a standardized distance from the surgical site and irradiation was performed for a fixed duration immediately after surgery and on predetermined postoperative days. In Group II, low-level laser therapy was delivered using a diode laser operating at a wavelength and power output suitable for photobiomodulation. Laser irradiation was applied in a non-contact mode over the surgical area with standardized energy density and exposure time. Both groups received their respective therapies following an identical postoperative schedule to ensure consistency. All participants were prescribed uniform postoperative medications, including analgesics and antimicrobial mouth rinses and were instructed to refrain from using any additional adjunctive healing aids during the study period. Sutures were removed after one week and patients were recalled for follow-up evaluations at designated postoperative intervals. Postoperative healing was assessed using clinical parameters such as pain intensity, degree of inflammation, tissue edema and wound healing index scores. Pain was evaluated using a visual analog scale, while soft tissue healing was assessed using a standardized periodontal wound healing index. Clinical signs including erythema, edema and bleeding were also recorded. All assessments were carried out by a blinded examiner who was unaware of the group allocation. The collected data were subjected to statistical analysis using appropriate statistical software. Descriptive statistics were used to summarize the data, while inferential statistics were employed to compare healing outcomes between the two groups. Intergroup comparisons were performed using independent t-tests or Mann–Whitney U tests and intragroup comparisons over time were analyzed using paired t-tests or repeated measures analysis, based on data distribution. A p-value of less than 0.05 was considered statistically significant.

## Results:

All 100 participants completed the study, with no dropouts or postoperative complications reported in either group. Group I (LED therapy) and Group II (low-level laser therapy) each comprised 50 participants and baseline demographic and clinical parameters were comparable between the groups, indicating successful randomization. No statistically significant differences were observed at baseline for age, gender distribution, plaque index, gingival index, or probing depth (Table 1). Postoperative pain scores, assessed using the visual analog scale (VAS), demonstrated a significant reduction over time in both groups. However, participants in the LLLT group exhibited significantly lower mean VAS scores on postoperative days 3 and 7 compared to the LED group, suggesting superior analgesic effects of low-level laser therapy during the early healing phase. By postoperative day 14, pain scores in both groups were minimal and no statistically significant difference was observed (Table 2). Assessment of soft tissue healing using the periodontal wound healing index revealed progressive improvement in both groups at all follow-up intervals. On postoperative day 7, the LLLT group demonstrated significantly better healing scores compared to the LED group, characterized by reduced erythema, minimal edema and earlier epithelialization. Although both groups showed comparable healing by day 14, the mean wound healing index remained marginally higher in the LLLT group, indicating a trend toward faster tissue repair (Table 3). Clinical evaluation of inflammatory parameters, including erythema and edema scores, showed a significant reduction from baseline to postoperative day 7 and day 14 in both groups. Intergroup comparison revealed that the LLLT group exhibited significantly lower inflammation scores at day 7, while no statistically significant difference was observed at day 14, indicating that both modalities were effective in controlling postoperative inflammation over time (Table 4). Overall, both LED therapy and low-level laser therapy proved effective as adjuncts in enhancing post periodontal surgical healing. However, low-level laser therapy demonstrated superior outcomes in terms of early pain reduction, faster resolution of inflammation and improved wound healing during the initial postoperative period.

## Discussion:

The present study's findings showing enhanced postoperative healing with both LED therapy and low-level laser therapy (LLLT) align broadly with existing evidence that photobiomodulation modalities can positively influence periodontal and oral surgical wound repair. LLLT, in particular, has been repeatedly reported to accelerate soft tissue healing, reduce inflammation and provide analgesic benefits in periodontal contexts. Kohale *et al.* (2018) [11] found that LLLT applied adjunctively after periodontal surgery resulted in significantly improved wound healing scores and reduced patient discomfort compared with control sites, indicating accelerated tissue repair consistent with increased cellular activity and angiogenesis following laser irradiation. Beyond surgical periodontal applications, systematic reviews have confirmed the positive influence of low-level lasers on wound healing more generally. For example, meta-analytic data suggest that LLLT significantly accelerates re-epithelialization and reduces pain in surgical wounds, including periodontal tissues, particularly within the first week postoperatively, reinforcing the early benefits seen in the current LLLT cohort relative to LED therapy [12]. These findings are mechanistically supported by molecular studies showing that laser irradiation can upregulate fibroblast proliferation, modulate inflammatory mediators and enhance neoangiogenesis-all key processes in early wound healing phases. Although direct clinical comparisons between LED and LLLT in periodontal surgery are limited, several lines of evidence indicate that LED photobiomodulation also fosters wound healing and inflammatory modulation. Chen *et al.* (2020) [13] reported that adjunctive 660-nm LED irradiation during periodontal therapy improved clinical parameters relative to control sites, suggesting a beneficial effect of LED light on periodontal tissue repair. Additionally, da Rocha *et al.* (2024) [14] summarized that LED irradiation stimulates cell proliferation, migration and angiogenesis pathways that are fundamental to wound repair, which provides a biological basis for clinical improvements observed in our LED group. Comparative reviews of phototherapy modalities suggest that while both LEDs and diode lasers can exert positive effects on soft tissues, the magnitude and consistency of outcomes may differ depending on parameters and clinical context. Lesniewski *et al.* (2022) [15] concluded that both diode lasers and LEDs are effective phototherapy tools in periodontology and oral surgery, but the current literature is insufficient to conclusively favour one over the other, highlighting the need for more direct comparative trials such as the present study. At the preclinical level, systematic analyses of experimental wound models indicate that LLLT might produce quantitatively greater effects on certain healing metrics (e.g., blood vessel density and collagen deposition) compared with LED therapy, even though both modalities promote typical photobiomodulation responses. This nuanced evidence may help explain why, in our trial, LLLT demonstrated superior early pain reduction and inflammatory control, while LED therapy still produced substantial healing progress over the study period.

## Conclusion:

Both light emitting diode therapy and low-level laser therapy were effective adjunctive modalities in enhancing post periodontal surgical healing by reducing pain, inflammation and promoting soft tissue repair. Low-level laser therapy demonstrated superior early postoperative outcomes, particularly in terms of faster pain control and inflammatory resolution during the initial healing phase. Within the limitations of the study, LLLT may be considered a more efficient adjunct in the early postoperative period, while LED therapy remains a clinically effective and practical alternative.

## Figures and Tables

**Table 1:** Baseline demographic and clinical parameters of study participants

**Parameter**	**LED Group (n = 50)**	**LLLT Group (n = 50)**	**p-value**
Mean age (years)	39.8 ± 7.2	40.3 ± 6.9	0.71
Gender (Male/Female)	28 / 22	30 / 20	0.68
Plaque Index	1.12 ± 0.24	1.09 ± 0.21	0.54
Gingival Index	1.34 ± 0.29	1.31 ± 0.27	0.62
Probing Depth (mm)	5.6 ± 0.8	5.7 ± 0.7	0.58

**Table 2:** Comparison of postoperative pain scores (VAS) between groups

**Time interval**	**LED Group**	**LLLT Group**	**p-value**
Day 1	5.8 ± 1.1	5.5 ± 1.0	0.18
Day 3	4.1 ± 0.9	3.2 ± 0.8	<0.001
Day 7	2.3 ± 0.7	1.6 ± 0.6	<0.001
Day 14	0.6 ± 0.3	0.5 ± 0.2	0.21

**Table 3:** Periodontal wound healing index scores

**Time interval**	**LED Group**	**LLLT Group**	**p-value**
Day 7	3.6 ± 0.5	4.1 ± 0.4	<0.001
Day 14	4.4 ± 0.3	4.6 ± 0.2	0.06

**Table 4:** Comparison of postoperative inflammation scores

**Time interval**	**LED Group**	**LLLT Group**	**p-value**
Day 3	2.9 ± 0.6	2.4 ± 0.5	0.002
Day 7	1.8 ± 0.4	1.3 ± 0.3	<0.001
Day 14	0.6 ± 0.2	0.5 ± 0.2	0.19
